# Association between Toenail Magnesium and Type 2 Diabetes in Chinese Adults

**DOI:** 10.3390/nu9080811

**Published:** 2017-07-27

**Authors:** Jiguo Zhang, Huijun Wang, Zhihong Wang, Ji Zhang, Bing Zhang

**Affiliations:** National Institute for Nutrition and Health, Chinese Center for Disease Control and Prevention, No. 29 Nanwei Road, Beijing 100050, China; zhangjg@ninh.chinacdc.cn (J.Z.); wanghj128@gmail.com (H.W.); wangzh@ninh.chinacdc.cn (Z.W.); zhangji@ninh.chinacdc.cn (J.Z.)

**Keywords:** magnesium, type 2 diabetes, adults, Chinese

## Abstract

Previous studies have showed an inverse association between magnesium level and type 2 diabetes, but the results are inconsistent, and the evidence relates only to dietary and serum magnesium. Moreover, it is not certain how these findings are applicable to Asian people. This study was designed to examine the association between toenail magnesium and type 2 diabetes in Chinese adults. The sample was 5683 adults aged 18 years or older from the 2009 China Health and Nutrition Survey. We used hemoglobin A1c equal to or greater than 6.5% as the diagnostic criterion for type 2 diabetes. Inductively coupled plasma–mass spectrometry determined toenail magnesium. Mean toenail magnesium in participants with and without type 2 diabetes was 263.0 ± 170.9 and 282.3 ± 191.9 micrograms per gram, respectively. The multivariable-adjusted odds ratio for type 2 diabetes comparing the highest to the lowest quartile of toenail magnesium was 0.72 (95% confidence interval, 0.52–0.99). We found a statistically significant interaction between toenail magnesium and geographic region on the prevalence of type 2 diabetes (*p* for interaction = 0.009). Our findings suggest that toenail magnesium is inversely associated with the prevalence of type 2 diabetes. Promoting the intake of magnesium-rich foods may bring considerable benefits for the prevention of type 2 diabetes, especially in those at high risk.

## 1. Introduction

Diabetes is a serious and increasing public health problem that urgently needs to be addressed across the world, particularly in the developing countries [1]. China is now among the countries with the highest diabetes prevalence in Asia, and has the largest absolute disease burden of diabetes in the world [2,3]. In a report by the National Health and Family Planning Commission, in 2012 the prevalence of diabetes was 9.7% for adults 18 years and older: 10.2% for men and 9.0% for women. Diabetes has already become one of the most common chronic diseases in China [4].

Diet plays an important role in the development of type 2 diabetes [5]. Magnesium is an essential cofactor for enzymes involved in glucose metabolism [6], and has received considerable attention for its potential in improving insulin sensitivity and preventing type 2 diabetes [7,8,9,10]. An inverse association has been observed between magnesium and type 2 diabetes in Western populations. However, it is not certain whether these findings are applicable to Asian people. Some prospective studies have investigated the association between magnesium intake and diabetes risk in Asian populations, but the findings are inconsistent [11,12,13,14]. Moreover, the association was only related to dietary and serum magnesium. To our knowledge, no previous study has evaluated the relationship between toenail magnesium and type 2 diabetes.

To fill gaps in the literature, we investigated the relationship of toenail magnesium with type 2 diabetes in Chinese adults.

## 2. Materials and Methods 

### 2.1. Study Population

The China Health and Nutrition Survey (CHNS) was designed to examine how the social and economic transformation in China affects the health and nutritional status of the Chinese population. The CHNS used a multistage random-cluster process to draw the sample in nine provinces that vary in demography, geography, economic development, and public resources. We used data from the survey conducted in 2009, when blood and toenail samples were collected for the first time. There were 6377 eligible participants 18 years or older. We excluded 80 participants who were pregnant or breast-feeding, 152 diagnosed with myocardial infarction or apoplexy, and 462 with missing or invalid values on other variables of interest. The final analysis included 5683 participants with complete demographic, biomarker, toenail, and dietary data. All participants gave written informed consent for their participation in the survey.

The study was approved by the institutional review boards of the University of North Carolina at Chapel Hill and the National Institute for Nutrition and Health, Chinese Center for Disease Control and Prevention (No. 201524).

### 2.2. Assessment of Toenail Magnesium

To obtain more samples of toenails, participants were asked to let their toenails grow for two weeks then use stainless steel nail scissors to collect as much as possible of their 10 toenails. The participants collected the samples themselves, placed them in envelopes the investigators provided, and returned the envelopes to the investigators. The toenail specimens were sent to a Beijing laboratory for analysis.

Inductively coupled plasma–mass spectrometry is a powerful analytic tool commonly used for multielement determinations of macro and trace minerals, and was used to determine toenail magnesium.

### 2.3. Outcome Measure

The CHNS collected blood samples in the morning after an overnight fast. Glucose, hemoglobin A1c (HbA1c), and insulin were measured with standard procedures and strict quality control. We used HbA1c at or above 6.5% as the diagnostic criterion for type 2 diabetes. Compared with a single measure of glucose, HbA1c captures long-term glycemic exposure, and it has been shown to be reliable for diabetes diagnosis among Chinese people [15]. Laboratory analysis methods are described in detail elsewhere [16].

### 2.4. Other Relevant Variables

We used the Xylidyl Blue colorimetric method to measure serum magnesium concentrations in millimoles per liter (mmol/L). We followed standard operating procedures for the collection, processing, and storage of blood samples. The CHNS assessed dietary intake by collecting three consecutive 24-h dietary recalls. Interviewers were trained to use standard forms for administering the dietary recalls in household interviews. The participants were asked to report all foods and beverages consumed both at home and away from home [17]. We used the average intake of the three recalls for each individual.

A general information questionnaire collected participants’ age, sex, education, geographic region (north/south), living area (urban/rural), cigarette smoking habits, tea intake, alcohol intake, physical activity, and annual household income. Well-trained health workers who followed the reference protocol recommended by the World Health Organization conducted anthropometric measurements [18]. We used height and weight measurements to calculate body mass index (BMI, kg/m^2^). We measured waist circumference at the midpoint between the lower border of the rib cage and the iliac crest to the nearest 0.1 centimeter (cm).

### 2.5. Statistical Analysis

We categorized toenail magnesium measurements into quartiles based on the distribution in the whole population and used the quartiles to compare nutrient intake and other lifestyle factors. With logistic regression analysis, we calculated the odds ratio (OR) and 95% confidence interval (95% CI) for type 2 diabetes across the quartile categories of toenail magnesium. We calculated multivariate adjusted ORs by adjusting for potential risk factors of type 2 diabetes, including age, sex, education, geographic region, living area, smoking status, alcohol consumption, tea drinking, physical activity, annual household income, and dietary factors such as total energy intake (kilocalories per day, kcal/day) and intakes of calcium (mg/day) and fiber (g/day). We assessed the linear trend of association with a logistic regression model with a median variable of toenail magnesium in each category.

We determined the association between toenail magnesium and type 2 diabetes prevalence for subgroups defined by age, sex, education, geographic region, living area, smoking status, alcohol consumption, and BMI. We obtained *p* values for the interactions from likelihood ratio tests comparing models with and without the interaction terms.

For all statistical analyses, we used version 9.2 of the SAS software package (SAS Institute, Inc., Cary, NC, USA). We considered a *p*-value less than 0.05 statistically significant.

## 3. Results

### 3.1. Characteristics of the Participants

Table 1 shows the characteristics of the participants according to diabetic status. Mean toenail magnesium values were 263.0 ± 170.9 micrograms per gram (μg/g) in participants with type 2 diabetes and 282.3 ± 191.9 μg/g in those without type 2 diabetes. The overall prevalence of type 2 diabetes in the target population was 7.5%. Participants with type 2 diabetes were older and drank more tea, were more likely to live in urban areas and the northern region, were less likely to be highly educated, had higher BMIs and waist circumferences, had higher percentages of energy intake from fat, and had lower intakes of energy and percentages of energy intake from carbohydrates compared with participants without type 2 diabetes.

Participants in the highest quartile of toenail magnesium were more likely to be male, current smokers, and current alcohol drinkers; to live in the northern region; and to have higher waist circumferences, higher intakes of total energy and fiber, and higher percentages of energy intake from carbohydrates (Table 2). Toenail magnesium levels were inversely associated with high education, calcium intake, and percentage of energy intake from fat. Participants living in urban areas had lower mean toenail magnesium than did participants living in rural areas.

### 3.2. Association of Toenail Magnesium with Type 2 Diabetes

The multivariable-adjusted OR for type 2 diabetes comparing the highest to the lowest quartile of toenail magnesium was 0.72 (95% CI, 0.52–0.99) (Table 3).

We conducted sensitivity analyses to test the robustness of our findings. We found similar association when the definition of diabetes was based on fasting blood glucose concentrations at or above 126 milligrams per deciliter (data not shown). The multivariable-adjusted OR for type 2 diabetes comparing the highest to the lowest quartile of toenail magnesium was 0.67 (95% CI, 0.48—0.94).

We found a statistically significant interaction between toenail magnesium and geographic region and the prevalence of type 2 diabetes (*p* for interaction = 0.009). The inverse association between high toenail magnesium and the prevalence of type 2 diabetes was consistent for current smokers, participants living in urban areas and the northern region, and participants with a BMI of 24 or greater (Table 4).

The correlation coefficients between dietary magnesium and serum magnesium was 0.031 (*p* = 0.019). The correlation coefficients between dietary magnesium and toenail magnesium was 0.119 (*p* < 0.001). There is no significant correlation between serum magnesium and toenail magnesium (Table 5).

## 4. Discussion

This large cross-sectional study of Chinese men and women shows that participants in the highest quartile of toenail magnesium had a statistically significant decreased prevalence of type 2 diabetes compared with those in the lowest quartile after adjusting for age, sex, BMI, and other potential confounders. The results suggest an inverse association between toenail magnesium and type 2 diabetes in Chinese adults. Our finding is consistent with previous studies among Asian populations [11,12,14]. Four previous meta-analyses confirmed the inverse association between dietary or total magnesium intake and risk of type 2 diabetes [8,9,10,19]. In contrast, the Atherosclerosis Risk in Communities study and the Japan Public Health Center-based prospective study did not find such an association between magnesium intake and type 2 diabetes [13,20].

Our study also found that geographic region modified the relation between toenail magnesium and prevalence of type 2 diabetes. The inverse association between toenail magnesium and type 2 diabetes was evident only among participants who lived in the northern region. Previous research has showed differences in diabetes prevalence between the north and the south in China [21]. However, it is unclear what mechanisms link geographic region with type 2 diabetes. The possible explanation is that associated risk factors such as diet, lifestyle, and higher BMI and waist circumference are clustered in the North [22,23,24]. 

In our subgroup analyses, the significant inverse association between toenail magnesium and type 2 diabetes was observed primarily among participants with BMIs of 24 kg/m^2^ or higher, but not among those with lower BMIs, although the test for interaction was not statistically significant. Previous studies also have found more pronounced associations in overweight and obese individuals than in individuals of normal-weight [8,9,12,25,26]. It is considered that high magnesium intake may have greater effects on overweight individuals, who are prone to insulin resistance and are more susceptible to the effects of magnesium on improving insulin sensitivity [25]. However, one study reported that magnesium had a significantly protective effect on normal-weight persons as well as overweight individuals [14]. Further studies are needed to assess the effect modification by BMI. Moreover, the inverse association was most apparent among current smokers and participants living in urban areas, who are high-risk populations for diabetes [27,28,29,30].

We included fiber and calcium as potential confounders due to their correlations with magnesium intake and protective effects against diabetes [11,19,31,32]. When we controlled for fiber and calcium intakes in our analyses, the inverse association between toenail magnesium and type 2 diabetes persisted and remained statistically significant, suggesting that the association is independent of the effects of fiber and calcium intakes.

Magnesium plays an important role in carbohydrate metabolism, and it may influence the hormones that help control blood glucose levels [33]. A review has shown that magnesium deficiency could impair the insulin signal transduction pathway and may affect the interaction between insulin and insulin receptors [34]. Conversely, magnesium supplements appear to be useful in persons with type 2 diabetes to mend magnesium deficiencies and to improve insulin resistance, oxidative stress, and systemic inflammation [35].

Assessment of magnesium status is difficult, since approximately 99.0% of total body magnesium is in bone and intracellular in soft tissue, with approximately 0.3% in serum [36]. Human nails are largely constituted of keratin-rich proteins, which incorporate trace elements in proportion to their consumption and other exposures by various mechanisms including protein synthesis and chemical binding with sulfhydryl groups [37]. Concentrations of some minerals in toenails can be used as biomarkers of exposure. The relatively high degree of reproducibility of toenail magnesium over a period of 6 years suggests that a single sample may represent long-term exposure [38]. However, there is a need to validate the measurement of toenail magnesium and to demonstrate that the sample represents a period of either deficient or excessive magnesium intake [39]. Up to now, it is unclear how to interpret magnesium values in nails.

Our study provides a unique opportunity to examine the correlations among dietary magnesium, serum magnesium, and toenail magnesium. In our study, the correlation coefficient between dietary magnesium and serum magnesium was 0.031 (*p* = 0.019). The correlation coefficient between dietary magnesium and toenail magnesium was 0.119 (*p* < 0.001). There is no significant correlation between serum magnesium and toenail magnesium. It is difficult to assess the comparisons among dietary magnesium, serum magnesium, and toenail magnesium, because there is no gold standard for assessing magnesium status in the human body.

This study has several limitations. First, the results do not show the causal or resultant relationship between toenail magnesium and type 2 diabetes due to the cross-sectional data. Second, we cannot completely exclude the possibilities of residual confounding from unmeasured factors such as family history of diabetes, uncertain factors such as treatment methods, or inaccurately measured factors. Despite these limitations, this is the first study to reveal the relationship between toenail magnesium and type 2 diabetes in Chinese men and women using large survey data. The present study is useful in providing a better understanding of toenail magnesium as a biomarker of exposure and the effect on type 2 diabetes based on the current dietary and serum magnesium research.

In conclusion, our findings suggest an inverse association between toenail magnesium and type 2 diabetes among men and women, especially among those who live in the northern region of China. Further prospective studies are needed to better understand the relationships. As for public health implications, this study supports diet modification to increase consumption of magnesium-rich foods, such as whole grains, nuts, and green leafy vegetables, which may bring considerable benefits for the prevention of type 2 diabetes, especially in those at high risk.

## Figures and Tables

**Table 1:** Characteristics of the study population by diabetes status *.

	Diabetes	No Diabetes	*p*
N	429	5254	
Age (years)	58.5 ± 11.9	50.0 ± 14.4	<0.001
Female (%)	52.7	52.2	0.851
Urban (%)	43.4	28.4	<0.001
North (%)	61.5	46.2	<0.001
Education (high) (%)	7.0	11.5	<0.001
Current smoker (%)	26.1	28.4	0.315
Current alcohol drinker (%)	33.8	33.1	0.767
Tea drinker (%)	41.3	34.3	0.026
BMI (kg/m^2^)	25.7 ± 3.7	23.2 ± 3.3	<0.001
Waist circumference (cm)	90.8 ± 10.1	82.3 ± 10.1	<0.001
Toenail magnesium (µg/g)	263.0 ± 170.9	282.3 ± 191.9	0.026
Serum magnesium (mmol/L)	0.9 ± 0.1	0.9 ± 0.1	0.056
Dietary intake			
Energy (kcal/day)	2117.9 ± 685.0	2205.7 ± 676.1	0.010
Carbohydrate (% of energy)	51.2 ± 11.6	53.9 ± 11.8	<0.001
Fat (% of energy)	33.0 ± 10.4	31.4 ± 11.8	0.002
Fiber (g/day)	12.2 ± 7.1	11.9 ± 8.3	0.537
Calcium (mg/day)	393.0 ± 207.1	395.1 ± 262.1	0.844
Magnesium (mg/day)	293.7 ± 110.3	301.0 ± 118.8	0.187

* Values are mean ± standard deviation for continuous variables and percentage for categorical variables.

**Table 2:** Characteristics of the study population according to quartiles (Q) of toenail magnesium *.

	Quartile of Toenail Magnesium				
	Q1	Q2	Q3	Q4	*p* ^†^
Median (μg/g)	129.5	198.9	271.9	453.6	
Age (years)	49.8 ± 14.7	51.6 ± 13.9	50.6 ± 14.5	50.5 ± 14.6	0.730
Female (%)	56.5	53.9	48.4	50.2	<0.001
Urban (%)	45.1	36.0	25.2	12.0	<0.001
North (%)	29.7	42.9	55.6	61.3	<0.001
Education (high) (%)	16.2	13.1	10.5	4.9	<0.001
Current smoker (%)	24.6	28.3	30.3	29.6	0.001
Current alcohol drinker (%)	30.3	31.8	34.7	35.8	0.001
Tea drinker (%)	36.5	36.0	36.3	30.3	0.030
BMI (kg/m^2^)	23.2 ± 3.4	23.5 ± 3.4	23.6 ± 3.5	23.3 ± 3.5	0.720
Waist circumference (cm)	81.6 ± 10.5	83.2 ± 10.2	83.8 ± 10.3	83.2 ± 10.2	0.001
Serum magnesium (mmol/L)	0.9 ± 0.1	0.9 ± 0.1	0.9 ± 0.1	0.9 ± 0.1	0.824
Dietary intake					
Energy (kcal/day)	2115.5 ± 618.4	2143.5 ± 673.3	2238.9 ± 703.6	2298.2 ± 694.8	<0.001
Carbohydrate (% of energy)	51.1 ± 11.7	51.9 ± 11.7	54.3 ± 11.3	57.6 ± 11.5	<0.001
Fat (% of energy)	33.5 ± 10.3	32.7 ± 10.3	30.8 ± 9.9	29.1 ± 15.1	<0.001
Fiber (g/day)	10.8 ± 6.7	11.8 ± 10.3	12.3 ± 8.0	13.0 ± 7.2	<0.001
Calcium (mg/day)	401.1 ± 256.7	402.3 ± 268.8	395.0 ± 251.2	381.2 ± 255.9	0.018
Magnesium (mg/day)	281.8 ± 104.5	289.9 ± 116.7	307.1 ± 125.6	323.0 ± 120.7	<0.001

* Values are mean ± standard deviation for continuous variables and percentage for categorical variables. † *p* for trend was calculated from a linear regression analysis for continuous variables and Mantel–Haenszel χ^2^ for categorical variables.

**Table 3:** Association between toenail magnesium and type 2 diabetes in the study population *.

	Quartile of Toenail Magnesium				
	Q1	Q2	Q3	Q4	*p* ^†^
Type 2 diabetes (%)	8.0	8.2	7.7	6.2	
Model 1	1.00 (reference)	0.96 (0.73–1.27)	0.93 (0.70–1.23)	0.73 (0.54–0.98)	0.026
Model 2	1.00 (reference)	0.89 (0.67–1.18)	0.84 (0.62–1.13)	0.72 (0.52–0.99)	0.053
Model 3	1.00 (reference)	0.89 (0.66–1.18)	0.84 (0.62–1.13)	0.72 (0.52–0.99)	0.051

* Values are odds ratio ORs (95% confidence interval CI). Model 1 is adjusted for age (continuous), sex (male or female), and education (low, medium, and high). Model 2 is further adjusted for geographic region (north or south), living area (urban/rural), annual household income per family member (continuous), physical activity (continuous), current smoker (yes/no), current alcohol drinker (yes/no), tea drinker (yes/no), and BMI (continuous). Model 3 is additionally adjusted for total energy intake (continuous), fiber (continuous), and calcium (continuous). † *p* for trend was calculated using the median value of each quartile as a continuous variable.

**Table 4:** ORs for type 2 diabetes comparing the highest to the lowest quartile of toenail magnesium in selected subgroups.

	Quartile of Toenail Magnesium					
	*n*	Q1	Q2	Q3	Q4	*p* for Interaction
Subgroups						
Age						0.452
18–44	1933	1.00 (reference)	0.60 (0.28–1.29)	0.42 (0.19–0.95)	0.44 (0.20–1.00)	
45–59	2207	1.00 (reference)	0.82 (0.51–1.31)	0.92 (0.57–1.47)	0.79 (0.47–1.31)	
60–	1543	1.00 (reference)	1.12 (0.74–1.69)	0.98 (0.62–1.53)	0.81 (0.49–1.34)	
Sex						0.612
Male	2714	1.00 (reference)	0.85 (0.56–1.29)	0.88 (0.57–1.35)	0.65 (0.40–1.05)	
Female	2969	1.00 (reference)	0.97 (0.65–1.44)	0.91 (0.60–1.38)	0.82 (0.52–1.28)	
Living area						0.210
Urban	1679	1.00 (reference)	0.93 (0.60–1.43)	0.39 (0.24–0.64)	0.61 (0.38–0.98)	
Rural	4004	1.00 (reference)	1.31 (0.89–1.95)	1.18 (0.78–1.78)	0.97 (0.64–1.49)	
Geographic region						0.009
North	2692	1.00 (reference)	0.71 (0.50–1.01)	0.68 (0.47–0.99)	0.52 (0.34–0.79)	
South	2991	1.00 (reference)	1.11 (0.70–1.76)	0.97 (0.60–1.58)	1.30 (0.79–2.15)	
Education						0.841
Low	2481	1.00 (reference)	0.82 (0.55–1.22)	0.90 (0.59–1.36)	0.71 (0.45–1.11)	
Middle	2568	1.00 (reference)	0.68 (0.43–1.06)	0.68 (0.43–1.08)	0.65 (0.40–1.08)	
High	634	1.00 (reference)	1.99 (0.68–5.85)	0.85 (0.24–3.01)	1.27 (0.36–4.45)	
Smoking						0.277
Yes	1603	1.00 (reference)	0.69 (0.40–1.21)	0.76 (0.43–1.34)	0.43 (0.22–0.84)	
No	4080	1.00 (reference)	1.02 (0.73–1.42)	0.88 (0.62–1.25)	0.88 (0.60–1.29)	
Alcohol						0.225
Yes	1884	1.00 (reference)	0.71 (0.43–1.18)	0.88 (0.54–1.45)	0.58 (0.33–1.01)	
No	3799	1.00 (reference)	1.10 (0.77–1.56)	0.88 (0.60`1.28)	0.88 (0.59–1.32)	
BMI						0.568
Normal <24 kg/m^2^	3345	1.00 (reference)	0.89 (0.54–1.46)	0.96 (0.58–1.61)	0.95 (0.55–1.64)	
Overweight >=24 kg/m^2^	2338	1.00 (reference)	0.90 (0.63–1.27)	0.80 (0.56–1.16)	0.62 (0.41–0.93)	

**Table 5:** Correlations of dietary magnesium, serum magnesium, and toenail magnesium.

	Dietary Magnesium	Serum Magnesium	Toenail Magnesium
Dietary magnesium	1.000	0.031, *p* = 0.019	0.119, *p* < 0.001
Serum magnesium	0.031, *p* = 0.019	1.000	0.020, *p* = 0.126
Toenail magnesium	0.119, *p* < 0.001	0.020, *p* = 0.126	1.000

Adjusted for age, sex, and BMI.
